# News and Miscellany

**Published:** 1900-08

**Authors:** 


### News and Miscellany.

**New Orleans Polyclinic.**—Physicians will find the Polyclinic an excellent means for posting themselves upon modern progress in all branches of medicine and surgery. The specialties are fully taught, particularly laboratory work. Fourteenth annual session opens November 12, 1900. For further informarion address Dr. Isadore Dyer, Secretary, New Orleans Polyclinic, New Orleans.

**Dr. T. J. Bennett,** this city, is visiting his parents in Wyoming.

**Dr. G. C. McLeod** has removed from Buckholtz to Ad. Ran, Milam county, Texas.

**Dr. A. N. Denton,** Editor of the *Texas Medical News*, is in New York attending the Polyclinic and Post Graduate.

**Professors** J. W. McLaughlin and S. M. Morris, of the Texas Medical College are summering at Colorado Springs, where they have opened an office.

**A few copies** of Dr. Daniel's book, "Recollections of A Rebel Surgeon" left. Price delivered, $1.10 If local check be sent add ten cents foı collecting.

**Dr. W. H. Way,** the popular eye, ear, nose, and throat specialist, of Austin, has recovered from a recent protracted spell of sickness and can be found at his office every day, as formerly.

**The Journal** has a paper on "Alcoholism," which was sent by the Sec. Brazos Valley Medical Association. The name of the author is not given. If the author sees this he will please drop us a line.

---

**Dr. E. D. Capps,** of Ft. Worth, left the 16th of July for Chicago and New York, to pursue post graduate work on the eye, ear, nose, and throat. The Doctor will be gone six weeks or two months, returning to Ft. Worth about September 1st.

---

**They "Are not Afraid."**—Dr. Elbert Wing, of the N. W. University Medical School, Chicago, who has recently visited Hong Kong writes us that the Americans in Hong Kong do not mind tne plague, which is endemic there, any more than adults do measles in this country.

---

**A writer** in the *Texas Medical News* says that the smallpox now prevailing amongst the negroes in Texas is not smallpox, but *Yaws*, and that he cures every case he sees. Vaccination appears to prevent it, and to arrest the spread in a community. That ought to settle the diagnosis.

---

**Battle & Co.,** St. Louis, will send you free, on request, a colered lithograph called "Gout." It is a copy of a drawing published in 1799, by Humphrey, London. No advertisement on the picture. It is the author's quaint conception of what gout looks like. Good picture to frame for office.

---

**Notice.**—All Surgeons, Assistant Surgeons, Acting Assistant Surgeons or Contract Surgeons, and Hospitsl Stewards, who served in the Army or Navy of the late Confederate States, will please send their postoffice address to Deering J. Roberts, M. D., Secretary Surgeons' Association, C. S. A., Nashville, Tenn.

---

**The Journal of Surgical Technology** is the title of a new periodical, to be published monthly, beginning July 1, 1900 It will be devoted to the consideration of the technic of surgical procedures, at a subscription price of $1.00 a year. Valuable premiums are offered with the first subscriptions. Address the Technique Publishing Co., 404 East 14th St. New York, N. Y., for sample copy.

---

**The East Texas Medico-Chirurgical Society** held its second meeting at Palestine, Texas, July 5th and 6th, (ult.) There was a large attendance and an interesting program was carried out. The Texas Medical Journal was unanimously adopted as its official organ. A number of the papers read will appear in the next issue. Dr. S. R. Burroughs of Buffalo is President, and Dr. E. E. Guinn, of Rusk, is Secretary.

**The American** Association of Obsteiricians and Gynecologists will hold its thirteenth annual meeting in the Assembly room of the Galt House, Louisville Ky., Tuesday, Wednesday and Thursday, September 18, 19 and 20, 1900, under the presidency of Dr. Rufus Hall, of Cincinnati, O.

The titles of papers are announced in the order of their reception. The permanent program will be classified and issued about August 25, after which date no further titles can be added or changes made in the printed program.

A cordial invitation is extended to the medical profession to attend the several scientific sessions of the association.

---

**Notice.**—To members of the Texas State Medical Association: Having received letters from various members as to errors in the minutes of the Waco meeting of the Association as published in the TEXAS MEDICAL JOURNAL and *Texas Medical News*. I beg to state for your information that I did not edit the minutes, and am not responsible for such reported error. A carbon copy of the minutes was furnished the Journals by my permission. I wish also to state in explanation of the delay which will occur in the issue of the transactions, that the same is due to the failure of the stenographer to comply with his agreement to send in the minutes and discussions within reasonable time. Fraternally yours,

H. A. WEST, Sect'y.

---

**Attention is Called** to the following new advertisements which appear in this issue:

Medical Department, University of Tennessee. Write to Dr. Paul F. Eve, Nashville, for catalog.

Medical Department, University of Texas, Galveston. Dr. H. P. Cooke, Dean, Galveston, or J. M. Lomax, Registrar, Austin, for all about the University.

Beaumont Hospital Medical College, St. Louis; Dr. J. T. Larew, Dean, for information.

Northwestern University Medical School, Chicago; Dr. Elbert Wing, Chairman of Committee.

"Viskolein," the Maltbie Chemical Company, 221 Fulton Street, New. York.

New Orleans Polyclinic, New Orleans; H. C. Smith, Secretary.

---

**Ex-Judge Abram H. Dailey,** a former President of the Medico-Legal Society, and a diligent student of the science of medical jurisprudence, when asked to send his views in reply to the following question:

What has been, in your opinion, the most notable advance in that branch of forensic medicine, with which you are familiar, during the nineteenth century? replied as follows:

My answer is, that, in my opinion, that which has prevented the inception and spread of disease, thorough sanitary laws, based upon a knowledge of the causes of disease and the removal of the same is the most notable.

The whole civilized world is interested in that subject. Medical and legal minds have everywhere been directed to it, and through their efforts legislative action has been taken, [except in Texas.—Ed.] and thereby all the civilized people enjoy a sense of security against the ravages of contagious diseases that they have never before experienced. The future is sure to increase this safeguard.—*Medico Legal Journal*, June.

---

**The Journal** has on file and will publish in succeeding issues the following original papers:

Post-partum Hemorrhage, Dr. L. Merriwether, Grapeland (E. Tex. Med-Chir. Sec.)

Scarlatina, or "Something Similar." Dr. D. T. Evans, Jewett. (E. Tex. Med-Chir. Sec.)

General Medicine, Dr. J. H. Joyce, Buffalo. (E. Tex. Med-Chir. Sec.)

Report of Chairman Sec. Obstetrics and Gynecology, Dr. A. R. Kuykendal. (Cent. Tex. Med. Ass'n.)

Preparation of Lying-in patient and Management of Normal Labor. Dr. J. H. Alexander, Troy. (Cent. Tex. Med. Ass'n.)

Treatment of The Insane; Dr. F. R. Ross, As't. Phys., Insane Asylum, Austin. (Cent. Tex. Med. Ass'n.)

Hysteria; Dr. M. S. McElharmon, Belton. (Cent. Tex. Med. Ass'n.)

Tretament of Hernia; Dr. H. A. Engelhardt, Houston.

The Old Man Must Go; Our Dream; Dr. F. M. Davis, Houston.

Report of Chairman, Section on Surgery, South Tex. Med. Ass'n; Dr. J. S. Price, Beaumont Texas.

Neurasthenia; Dr. Virginius W. Gayle, Kansas City, Mo.

