# Correction

**Published:** 1900-09

**Authors:** 


**Correction:**—The paper on alcoholism mentioned in our last issue, and the author of which is unknown to us, was *not* sent by the Secretary of the Brazos Valley Association, and was *not* read before that association, as stated by us. The paragraph was written some months ago and stuck in the pigeon hole for future use, and altho Dr. Briggs, the Secretary, in reply to our inquiry had informed us that it was not sent by him nor read at the time stated, I simply overlooked the matter and failed to correct it. Our apologies are tendered Dr. Briggs for thus unintentionally putting him in a false position.—DANIEL.

